# Does Green Governance Efficiency and Green Finance Polices Matters in Sustainable Environment: Implications for Public Health

**DOI:** 10.3389/fpubh.2022.861349

**Published:** 2022-03-14

**Authors:** Siyun Xu, Huiqin Zhu

**Affiliations:** ^1^Financial Innovation and Risk Management Research Center, Hebei Finance University, Baoding, China; ^2^Department of Business Administration, Jeonju University, Jeonju, South Korea

**Keywords:** green finance, green governance efficiency, generalized panel three-stage DEA, provincial, public health

## Abstract

Rapid and widespread changes in the environment and climate, such as rising temperatures, water and air pollution, floods, and droughts, disease vector migration are putting human health at risk. In this case, green governance is an essential driver for the restructuring of economic development and realizing a green technological revolution for sustainable development and its implications for public health. This article aims to explore the effects and interrelationships of green governance and green finance policies on sustainable development in various regions of China's from 2008 to 2018 using panel data estimation technique. The findings show that China's overall green governance index and green finance policies resulted in a substantial decrease in environmental pollution during the study time. Financial inclusion also be a factor to the reduction of CO_2_ emissions and has a positive influence on environmental security investment projects, according to our findings. China is on track to become a world leader in an enactment of green finance concept, and controllers must speed up the development of green finance products and strengthen financial institutions' ability to provide green credit. Policymakers should promote green governance and green fiancé to keenly play a part in environmental security projects that boost green spending while minimizing the procedural risk.

## Introduction

The industrial sector in China accounts for 34.3% of the country's GDP (1). It does, however, consume ~70% of the entire energy utilization of China and emits about 80% of the unwanted gases (2, 3). The Chinese government has taken substantial steps in recent years to fully utilize its manufacturing solid waste, treatment of sewage, and eliminate unwanted gases. In background, a complete evaluation of China's manufacturing and waste treatment functioning will uncover the country's previous losses and gains, allowing future policy adjustments and improvements to be made (4). Recently the China's industrial sector which is high energy and emission intensive in other words the environmental efficiency, green manufacturing, and green energy efficiency has taken much intention of scholars (5, 6). In 2004, scholars applied an input based data envelopment analysis (DEA) to investigate China's environmental effectiveness in industry, the findings shows that out of 30 China's provinces only five were environmentally efficient (7). From 1997 to 2008, a Malmquist index was used to examine energy effectiveness in China's district manufacturing parts, and it was discovered that technological advancement was the most important factor in increasing China's manufacturing energy efficacy. From 1998 to 2009, a non-radial DEA was used to examine China's regional environmental performance. They noted that during this time, the country's environmental efficiency increased by 58% in Industry sector, with high-tech advancement being the primary driver (8). From 1980 to 2010, a Luenberger index was used to find green output across China's manufacturing areas, demonstrating that China's industry has yet to find a path toward less-carbon, sustainable development (9).

The green economy is a key tool for achieving sustainable development and describes an effective path to it (10). Energy consumption of china constantly rising and its becoming world largest consumer of the world due to rapid urbanization and industrial development (11–14). However, the environment has been severely harmed (15, 16), and effluence has increased (17, 18). As a result, in the face of economic change, China has realized that for economic benefits ignoring the principles of green economic development causes unsustainability. Green governance efficiency (GGE) is a major element for achieving sustainable growth because it combines environmental sustainability and economic development (19). Under resource constraints, GGE is defined as achieving higher economic output with minimal environmental cost. A thorough examination of China's GGE reveals the attribute of the country's economic development and, as a result, contributed significantly to worldwide long-term growth.

Despite the fact that in 2013 the coal consumption of China peaked, in 2014 fossil fuels quiet consisted of 87.67% of the country's whole energy consumption, and China's coal utilization accounted for 52% of world carbon emissions (20). China, in particular, exceeded the United States in emitting carbon in 2006 and had become the world's greatest carbon emitter and in 2010 China become the world's largest energy consumer, consisted of roughly 28% of global CO_2_ emissions. Since 2015, the consumption of coal in China has reduced although coal energy was accounting for 70% of whole energy consumption in 1978 and in 2017 it roughly account for 21% of whole global CO_2_ emissions (21). Furthermore, energy demand in China continues to rise and has consistently been the maximum in the Globe. Due to its “rich coal, meager petroleum, and little gas” natural resource allocation, China's energy structure is coal-based, with a low fraction and minor function of qualified energies such as gasoline and natural gas compared to the global average. Coal causes a much high degree of CO_2_ emission coefficient than gasoline or natural gas. Given that coal's dominant position in China's energy structure is unlikely to change anytime soon, increased pressure on CO_2_ emissions and quality control of air is expected to escort China's continued regional and economic development (22).

Green governance is a developing field that is gaining more and more attention from academics and is gradually becoming a focal point of policymaking in various governments worldwide. But the development of green governance is frequently hampered by a lack of clarity in the definition of various subjects' responsibilities and the desire for capital on the part of governments, businesses, and individuals in general (23–26). This has resulted in a current practice of green governance that is often limited to the spontaneous production of green goods and services as well as the management of green supply chains and administration of green administration of one single subject. For green governance to be effective, organizational boundaries must be broken, relationships between multiple governance subjects must be coordinated, a synergetic mechanism based on trust and contract must be built, and a governance mode of open innovation must be explored in order to achieve the goal of sustainable development for both humans and nature.

As a result, this manuscript first develop a green governance estimation index system to scientifically measure the effectiveness of green governance in every province. The resource contribution adjustment pathways of green governance is then clarified based on the redundancy analysis. Finally, the panel quantile regression is applied to investigate the impact of green governance and green finance on environmental sustainability. Our contribution also includes a three-stage DEA model-based empirical evaluation of Chinese provinces.

Rest of the paper is designed as follows: Section Literature Review discuss the green governance and environmental quality literature; Section Method and Data Sources presents the data and methodology; Section Results and Discussion presents the results analysis; and finally Section Conclusions and Policy Implication conclude the study and provide policy recommendation for decision maker.

## Literature Review

### Green Governance and Environmental Quality

Green governance research is still scarce, and most of it is based on ecological theory. Because of the various research objectives, scholars' definitions of green governance are also quite different. There are three types of definitions available. To begin with, governance is confused with management. Green governance, according to Rajesh (27), is the government's idealistic, tactical, and involved sustainable natural resource administration. Second, governance and governance structure are synonymous. Green governance, according to Nansikombi et al. (28), consists of five structures: norms, common objectives, involvement, communication, and resources. Finally, sustainable development is defined as green governance. Green governance, according to Omri and Ben Mabrouk (29), is a long-standing social, economic, and ecological sustainability.

While governance is a multidisciplinary idea, researchers' interpretations of its meaning and characteristics are generally consistent. Scholars agree that governance is an organizational ordering which is used to resolve disputes among investors, enabling them to take combined actions in order to reach systematic results. Synchronization, collaboration, and systematic decision-making are clearly the hallmarks of good governance. According to Yin et al. (30), governance has the subsequent characteristics: (1) Governance is distinct from management and is based on synchronization rather than control. (2) Governance comprises a number of interconnected parties, including the public and private sectors, as well as economic organizations. (3) Governance emphasizes the importance of balancing benefits and making systematic decisions. (4) Governance is a uninterrupted contact with the goal of maintaining relationship continuity.

The term “green” has a lot of different meanings. Green is both a sign of life and the color of nature's background. As a result, green is frequently used to refer to social, economic, and ecological procedures, that can also signify humanity's association with nature (31). In the current state of mankind's relationship with nature, the following fragility factors have been observed. For starters, because nature is a silent contributor, the association between nature and mankind is neither a harsh limitation nor a totally effective game law (32). Due to the typical principal–agent characteristics, there is a wide range of human opportunism, which necessitates the use of suitable approaches to defeat it. Second, green is the most equitable public good. A healthy eco-friendly environment is a critical component of human survival and development. The destruction of eco-friendly environment has direct influence on human manufacturing and life, necessitating the creation of a human fortune society, establishing strong regional and global collaboration, and allowing participants to share “green” in a reasonable and sensible way (33). Third, green activities have a high degree of externality, implying that they affect all aspects of the social, economic and ecological system (e.g., government, companies, known organizations, the nature, and community). In terms of decision-making, these subjects are autonomous and decentralized. Self-regard maximization can easily lead to irregularities in participant behavior and the overall goal. As a result, such an endeavor necessitates coordination of the various participants' interests, demands, and responsibilities.

Since ecological factors has got a global attention, green governance also has become the hot topic for scholars. It has become an global frontline concern. Green governance is the result of a four-stage evolution process that includes the conventional growth outlook, sustainable growth outlook, “green plus,” and green governance (34). Manhood lived in landscape with surprise and terror during the period of primitive evolution. Manhood exploited resources of nature and advanced to acquire ingredients and additional profits while the farming evolution. Despite the massive increase in productivity that resulted from the industrial revolution, tensions between mankind and nature grew during the industrial civilization. Many economists at the time used resource scarcity theory (i.e., the “traditional development view”) to investigate the association among economic growth and natural resources. la Cruz et al. (35) was primarily concerned with the interplay between population, environment and resources. If there is constant rise in population, he believes that it will grow at a symmetrical rate, while bodily resources will only grow at an arithmetic rate. If persons consistently to spend huge quantities of natural resources while disregarding their limited availability, the equilibrium between manhood, and nature will be destroyed, resulting in a catastrophic population reduction. Ju et al. (36) proposed the idea of “relative resource scarcity theory,” which states that technology can compensate for resource scarcity in a limited way. He believed that technological advancement could resolve the human-nature conflict. However, one point that cannot be overlooked is that technical advancement may result in ecological issues. Peng et al. (37) joined the opinions of Malthus and Ricardo, believing that the population and a country's natural resources, and capital should all stay stable at a distance from natural resource limits in order to avoid food shortages and the loss of natural beauty.

The global natural environment has gotten a lot of attention in recent years, and the global society has offered models like green economy, green growth, and green development. Apart from minor semantic differences, green economy, green growth, and green development are all applied interchangeably in reports from various international organizations (38). Pearce primarily offered the idea of a green economy in his volume Blueprint for a Green Economy, published in 1989, and recommended that the environment and the economy are intertwined. Moreover, this book only focuses the term “green economy” to refer to environmental strategies rather than recommending the model. The United Nations Environment Program (UNEP) presented the concept of green economy in 2007, and it is defined as “an economy that emphasizes people, nature, and the creation of high-paying jobs.” The definition was changed to “an economic development model that improves human welfare and social equity while greatly reducing environmental damage and ecological scarcity” by UNEP in 2011 (39). Green growth is defined as “an environmentally sustainable economic process to promote low-carbon development and benefit all members of society,” according to the United Nations' Asia-Pacific Economic and Social Committee (UNESCAP) ministerial conference on growth and environment in 2005. Green growth was further modified and deepened in 2011 by the Organization for Economic Co-operation and Development (OECD), which stated: “Green growth ensures that natural assets can continue to improve resources and environmental services in human society while also promoting economic growth and development.” Green growth, according to the World Bank, is defined as “achieving efficient, clean, and flexible production processes without slowing economic growth” (40). The term “green development” has still to be defined in a combined way. The World Bank and the Joint Research Group of the Development Research Center of the State Council believe that green development refers to “economic growth that is free of excessive use of resources, carbon emissions, and environmental damage, promoting growth by creating new green product markets, green technologies, green investments, and changing consumer and environmental behaviors,” according to “China in 2030: Building a Modern, Harmonious, and Creative Society.”

The current researches on green governance have the following attributes, in summary. Green governance has not yet been detached from researches on the development model, which represent that researchers use the term “development view” in its place of “governance” in the early stages. Related researches start to emphasize the synchronization between humans and nature during the “green plus” stage, though the game playing between humans and environment has still to be analyzed in the governance structure. From the standpoint of research hypothesis, the economic man hypothesis, which places “man” outside of nature, is gradually changing and evolving into the “ecological social economic man” hypothesis, which considers the interests of non-human life species and ecosystems (41). According to the evolution of research paradigms, traditional research does not adequately cover the connotation and extension of green governance and does not give sufficient intellectual and theoretical grounding. The economic and ecological features of green governance can be met simultaneously under an open innovation paradigm, which provides an effective model for resource reintegration and effective utilization (42). Green governance theories are presented, together with green governance innovation subjects, processes, and modes of implementation, as well as their consequences for green governance.

Previous studies have viewed eco-efficiency as a single procedure, concentrating on the businesses subdivision, which produces CO_2_ emissions directly, while ignoring the government's critical role. The government sector is important because it offers public commodities and services that help to create a productive environment. It is still unknown how the government sector influences the business sector or even total environmental efficiency. These studies misplaced focus segments for policy governance because they did not account for sectoral associations and relations within China's GGE.

### Green Finance and Environmental Quality

In light of the heightened worldwide efforts to prevent climate change, green finance has gotten a lot of attention recently. The acceptance of the UN Sustainable Development Goals and the Paris Climate Agreement were significant accomplishments for international organizations and national governments, demonstrating a stronger dedication to environmental sustainability (43). The Asian Development Bank (ADB) plays an important role in this regard, as it is involved in a number of projects aimed at ensuring environmentally sustainable development in the Pacific and Asia. Climate change finance is one of these initiatives, which is a joint project of the ADB and the Global Environment Facility (GEF). Climate mitigation finance is one of its components, which comprises the investing method that provides finances to achieve environmental sustainability in the country.

According to recent studies, green finance can improve environmental quality by supporting environmental regulations and reducing CO_2_ emissions. It may decrease fossil fuel utilization by 26%, resulting in a 12.4% reduction in CO_2_ emissions (44). Green finance is linked to a number of sustainable development goals, both directly and indirectly, that can be attained by encouraging private sector involvement in investment and green finance (45). Despite the fact that green finance is fully popular than always, few academics have inspected its impacts on environmental quality empirically. This raises the question of whether the growing trend in green financing exacerbates the conflict between environmental conservation and economic growth (46). Green Finance Because of this, the exact link between green finance and environmental degradation remains a mystery despite the importance of the subject. The purpose of this study is to provide empirical evidence to fill this gap (47). Stakeholders (organizations, governments, and regulators) seeking to benefit from environmental strategies may choose to participate in green finance on a tactical basis. Accepting the implications of green finance and the subtleties involved in improving environmental quality could thus be critical in persuading shareholders to use green finance.

Environmental consciousness among various participants has increased, according to previous research. The literature, in particular, sheds light on end-user-driven organizational environmental initiatives as well as pressure from monitoring entities and non-governmental organizations to proficiently craft their environmental actions and policies (48). The UN platform Convention on Environment Change also requires nations to take eco-friendly actions as a means of reducing pollution. Green finance appears to be critical to the success of these initiatives. As a result, moving from a growing economy to a green economy necessitates a commitment from a country's leadership to provide green financing. New stakeholder awareness and institutional setting for environmental problems are likely to lead regulators to explore for extra environmentally acceptable financial resources. Proactive environmental efforts will be necessary as new ways of obtaining funds arise in an effort to attain environmental legitimacy.

### Financial Inclusion and Environmental Quality

The employment of economic incentives to safeguard the environment is a common practice. There is a limit to how much environmental degradation may be tolerated by financial growth incentives (49). The environmental Kuznets curve was established by Grossman and Krueger (50) to describe the link between GDP, environmental degradation, and GDP. Financial development, unrestricted trade, energy use, and institutional effectiveness all contribute to the degradation of environmental practices. There is a lot of overlap in the way that financial inclusion affects the environment.

Either a net reduction in CO_2_ emissions or an increase in them may result from financial inclusion, depending on the situation. For green technology investments, financial products that give greater advantages at lower prices make it easier (51). Financial inclusion, on the other hand, enables companies and individuals to get access to more advantageous and cheap financial solutions. By expanding accessibility and affordability, as well as adopting more environmentally friendly practices that help reduce climate change, included financial systems assist the environment. Low-income communities cannot afford to overlook the need of financial inclusion. Clean energy solutions like solar microgrids, for example, are less expensive and generate considerably less CO_2_ than coal-fired power plants. However, farmers may lack the cash or financing to invest in these technologies (52). Major obstacles to the adoption of solar home systems in Ho Chi Minh City include a lack of funds, the government's financial help, and the availability of bank financing. Financial products and services that encourage the use of clean technology while also reducing CO_2_ emissions and the usage of fossil fuels can have environmental benefits.

## Method and Data Sources

### Method

The DEA method is a well-known numerical model that was proposed by (53) to measure efficiency. The DEA has recently become popular for quantitatively analyzing sustainability-related issues. DMUs are permanently a “black box” in traditional DEA models, though they could comprise a two step network in certain cases. Researchers have mostly established wide network-based DEA models to improved evaluate the efficacy of two-step systems and open this “black box.” Energy efficiency influence factors and measurement, the association between energy efficiency and environmental regulation, and the environmental impacts of environmental deregulation have all been studied in depth. The measurement of energy efficiency can be done using a variety of indicators (2, 24, 54–56). The most frequently used predictors are total factor and single factor energy efficiency (57). The first is a single-factor index that primarily reveals the association between economic output and energy consumption. Energy consumption intensity, also known as energy consumption per unit GDP, is most widely applied single factor index. Certain researches applied single-factor efficiency index to investigate the factors that influence energy efficiency, concluding that technological development, R&D spending, possession transformation, and business structure all have significant impact on energy efficiency.

GE has been the subject of numerous studies. The evaluation indicator system and the performance indicator system are the two most common approaches to measuring efficiency and methods for assessing efficiency. The parameter-based stochastic frontier analysis (SFA) and the non-parametric data envelopment analysis (DEA) method are the two major kinds of efficiency approaches. The DEA and SFA methods empirically find the efficiency of a managerial decision making unit with outputs and inputs, in contrast to subjective weighting indicators, which mostly comprise a extreme level of ambiguity in the assessment findings, and have thus been extensively used to measure the ecoefficiency of main global economies (58, 59). The SFA method, on the other hand, necessitates the specification of a production function and is inappropriate to quantitative measurements with multiple inputs and outputs (60).

When it comes to the association between energy efficiency and environmental regulation, some academics results shows that environmental regulation has raises the production cost of a company and reduces its competitiveness. Environmental regulations, as like emission standards and environmental taxes, in particular, compel businesses to reduce their output in order to reduce pollution emissions. The influence of environmental directives on the production effectiveness of comparatively adulterating businesses in the United States of America was investigated, and the food treating businesses was chosen as the study object. Environmental regulations reduced the food processing industry's production efficiency (61, 62). Inspite the rising publications on environmental regulation, decentralization, and cumulative efficiency, this area of study does have some restrictions. For starters, previous studies did not combine environmental regulation, decentralization, and energy efficiency into a single empirical and conceptual framework. Second, previous research ignored the link between environmental regulation and energy efficiency in various types of environmental decentralization, such as environmental management, environmental regulation, and environmental surveillance. Ultimately, in terms of research approaches, the majority of earlier studies used static research and linear analysis (63). Endogeneity issues arising from a mutual usual association between variables cannot be effectively addressed. Furthermore, ordinary panel data is used to evaluate the majority of the related studies. In fact, ignoring the possibility of spatial dependence between variables, The government's environmental regulations and the GGE have a certain spatial dependence.

#### Generalized Panel Three-Stage DEA Model

Green governance efficiency has got much attention from scholars as well as government departments due to increasing issue of environmental sustainability, increasing demand of energy and environmental degradation due to global warming (64). Most existing energy efficiency indices, on the other hand, do not take into account undesirable outputs like Sewage water, pollutant emissions, and solid waste creation. Taking into account the concept of sustainable growth and previous studies, this paper calculates a green governance efficiency index (GGE) for energy efficiency, which takes into account all unwanted output. When the unwanted production (pollution) is maintained to a low under the provided desired output parameters, GGE is defined as the ratio of the theoretical minimal energy input to the actual input point. Traditional data envelopment analysis (DEA) methods typically use radial and angular metrics to determine the efficiency of decision-making units (DMU). As a result, a traditional DEA technique can only begin from the position of input or from output, making it challenging to fully consider input and output relaxation. At a time, when a specific amount of all inputs decrease or increase, the output will also decrease or increase with same amount, is the only way to measure inefficiency. The relaxation improvement element is not represented in the efficiency assessment of the standard DEA model for faulty decision-making units, besides the equal fraction improvement part, and the real input-output never becomes equivalent to percentage change. Grounded on this, Relaxation variables were introduced straight into the impartial function, and non-radial and non-angular slacks-based measure (SBM) methods were proposed. Simultaneously, the inefficiency situation was calculated from the input and output angles, preventing the impact of the angle and radial selections. The fundamental SBM method output is set to the projected output, overlooking the exterior negative impacts of the production process on the environment.

Assuming the presence of input stability of green governance, the input-oriented variable returns to scale DEA model, i.e., the generalized BBC-DEA model, is chosen in this paper. Let the number of decision-making units (DMUs) be *n* and each DMU form $\bar{n}$ sample units (SU) at *t* time periods, i.e., $\bar{n}=nt$. The model is specified as:


(1) $$\begin{array}{l} Min\left[\theta -\varepsilon (\sum_{i=1}^{m}S_{ip}^{-}+\sum_{i=1}^{m}S_{ip}^{+})\right]\\ s.t.\{\begin{array}{c} \sum_{i=1}^{\bar{n}}{\bar{x}}_{ij}\lambda_{j}+S_{ip}^{-}=\theta_{p}x_{ip},i=1,\ldots m\\ \sum_{i=1}^{\bar{n}}d{\bar{y}}_{rj}\lambda_{j}-S_{ip}^{+}=y_{rp},r=1,\ldots s\\ \sum_{i=1}^{\bar{n}}\lambda_{j}=1\\ \lambda_{j}\geq 0,j=1,2,\ldots n,S_{ip}^{-}\geq 0,S_{ip}^{+}\geq 0 \end{array} \end{array}$$

Where each decision unit has *s* outputs and *m* inputs. The *i* input of the *p* decision unit is *x*_*ip*_ and the *r* output is *y*_*rp*_. The *i* input of the *j* sample cell is ${\bar{x}}_{ij}$ and the *r* output is ${\bar{y}}_{rj}$. θ_*p*_ is the efficiency value to be evaluated. $S_{ip}^{-}$ and $S_{rp}^{+}$ are slack variables for the input and output indicators, respectively. *d* is a mobility factor to characterize the progressivity of the system. ε is a non-Archimedean infinitesimal. λ_*j*_ is the weighting variable.

#### Generalized Panel DEA Model Adjusted for Input Variables

The adjusted input variables from Equation (3) are substituted into model (1) and recalculated to obtain the settled green governance efficiency value θ_*j*_ for every province. Based on the investigation of the efficiency frontier surface, the optimum input is revealed, the actual input is compared with the optimum input to obtain the input redundancy value, the forecast examination of the input variables is realized and the direction of improvement of the input variables is confirmed. The *i* optimum input value ${\hat{x}}_{ij}$ for the *j* sample cell can be obtained by the DEA projection formula:


(2) $$\begin{array}{l} {\hat{x}}_{ij}=\theta_{j}{\bar{x}}_{ij}-S_{\bar{ij{}^{\circ}}} \end{array}$$

Based on Equation (4), the redundancy value Δ*x*_*ij*_ for the *i*th input of the *j*-th sample cell is calculated as:


(3) $$\begin{array}{l} \Delta x_{ij}={\bar{x}}_{ij}-{\hat{x}}_{ij}=\left(1-\theta_{j}\right){\bar{x}}_{ij}+S_{\bar{ij{}^{\circ}}} \end{array}$$

By referring to Li Wei'an's green governance evaluation index system for company operations, the basis of this paper's research is improved: the government green governance evaluation index system. The details are shown in Table 1. To determine total factor energy efficiency of China under environmental constraints, this research uses a panel data set of 30 provinces and a three-stage DEA approach. Inputs, undesirable production and desirable outputs, are all included in the approach. The inputs are categorized as follows: Investment on Environmental governance, number of employees and waste gas discharge. A variable of the gross domestic product is one of the desired outputs (GDP) and the total CO_2_ emissions at provincial level is the undesirable output.

**Table 1:** The input and output indicators for green governance efficiency.

		**Indicator**	**Definition**
Inputs	Capital input	Investment on Environmental governance	The annual environmental governance fee of the wastes
	Labor input	Number of Employees	The average number of employees per year for environmental protection
	Waste discharge	Waste gas discharge	The total amount of waste gas and wastewater discharged
Outputs	Expected output	GDP	Gross provincial product
	Undesirable outputs	CO_2_ emissions	The total CO_2_ emissions at provincial level

### Econometric Technique

#### Cross Sectional Dependency

Cross-sectional dependency in panel data analysis might lead to incorrect estimation results (65). The Pesaran CD test, which is valid for either a constant T or a constant N, will be used in this investigation. Equation (10) is used to calculate the Pesaran CD statistic.


$$\begin{array}{l} CD=\sqrt{\frac{2T}{N\left(N-1\right)}}\;\left(\overset{N-1}{\underset{i=1}{\sum }}\overset{N}{\underset{J=i+1}{\sum }}{\hat{\rho }}_{ij}\right)\;N\left(0,1\right) \end{array}$$

Where ${\hat{\rho }}_{ij}$ the error term pairwise-correlation sample estimate. When *T* → ∞ and *N* → ∞ the refers to a normal distribution.

### Data Sources

On the basis of the availability of data and its analytical usefulness, this research focuses on China's 30 provinces between 2008 and 2018. Over the period 2008–2018, annual data on CO_2_ emissions, green finance, financial inclusion, natural resources, human capital, and remittances in 30 Chinese provinces was gathered online from the China Statistical Yearbook (2008–2019), China Energy Statistical Yearbook (2008–2019), China Environmental Quality Report (2008–2019) and China Environmental Statistics Yearbook (2008–2019). For the missing data, the post-evaluation was calculated using the average annual growth rate as a basis for comparison. Industrial Bank Co., Ltd. (Fujian, China) began its green finance practice in 2005, but it was the publication of Opinions on Implementing Environmental Policy and Regulations to Prevent Credit Risk in 2007 by the China Banking Regulatory Commission, the People's Bank of China, and the State Environmental Protection Administration that marked the official beginning of China's green finance practice. As a result, the data used in this study was collected starting in 2008. Table 2 presents the descriptive statistics of study variables.

**Table 2:** Descriptive statics.

**Variable**	**Explanation**	**Min**	**Max**	**Mean**	**SD**
CO_2_	Carbon emission	0.728	15.297	12.080	1.363
GGEI	Green governance efficiency index	0.38	1	0.737	1.212
FI	Financial inclusion	2.976	6.000	5.183	0.540
GF	GDP per capita	9.005	13.709	11.131	0.623
NR	Natural resources	0.728	15.694	10.686	1.814
HC	Human capital	2.522	4.722	4.035	0.285
RMT	Remittances	0.000	1.000	0.214	0.423

## Results and Discussion

### Green Governance Efficiency in China

**Figure 1:**
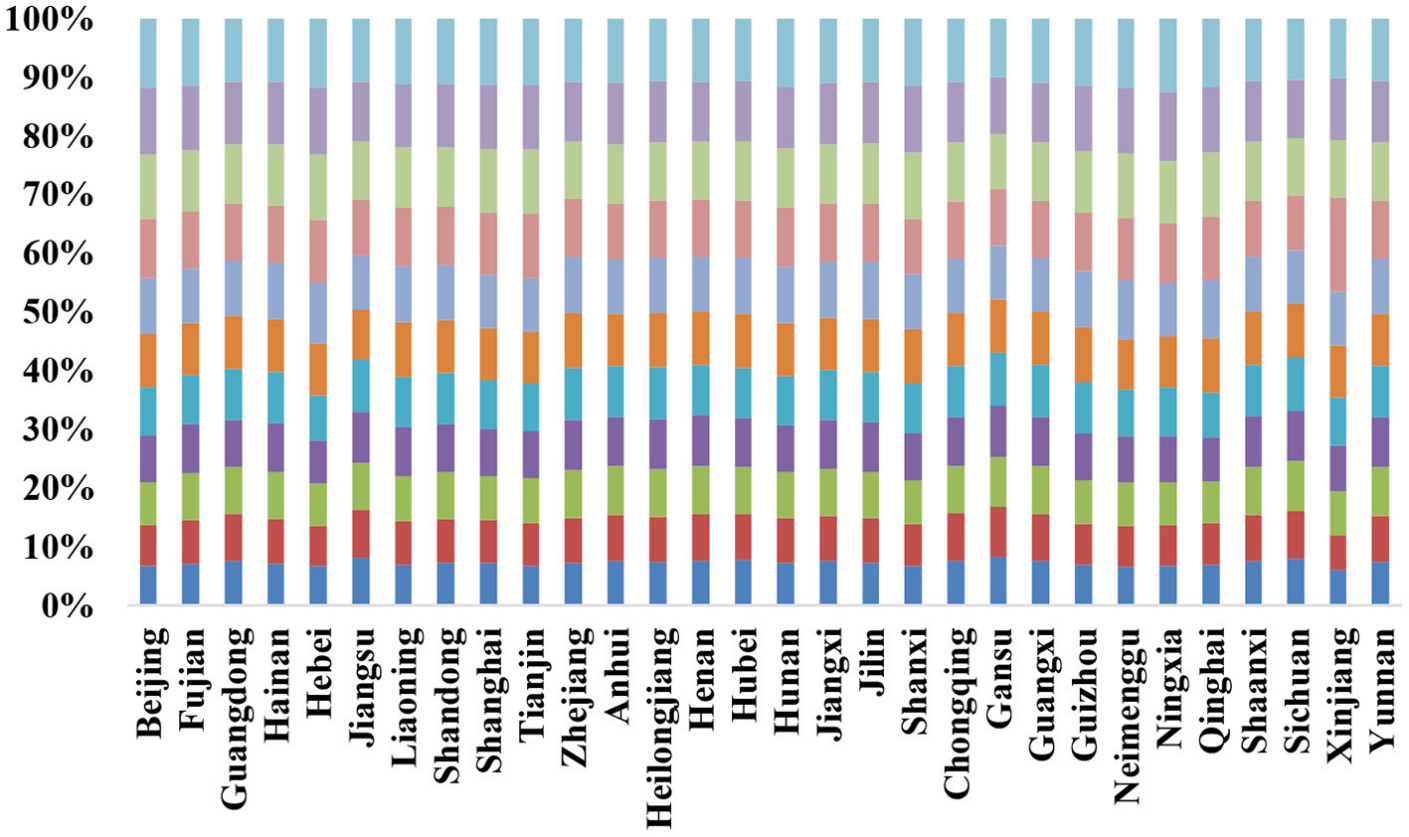
Results of green governance efficiency before adjustment.

A low level of efficiency was evident in Table 3 and Figure 1. Only 0.56 was the average between 2008 and 2018. When compared to advanced regions in China, this is only a basic level of development. Green governance efficiency will be lower when compared to more advanced regions of the world. Beijing had the highest average efficiency of 1.19 out of the 30 provinces, while Xinjiang had the lowest average efficiency of 0.20. It was found that Beijing and Shanghai were the provinces with the highest average green governance efficiency values while Guangdong and Hunan were the provinces with the lowest average green governance efficiency values. This shows the environmental governance investment of different provinces in China. Therefore, the level of efficiency varies significantly. Most academics have come to the same conclusion. For instance, according to Li et al. (66), the green governance efficiency of different Chinese provinces is spatially dependent and the neighbor effect is significant. The spatial distribution of governance efficiency in China is unbalanced. Economically advanced provinces in the east have higher rates of governance efficiency, while economically developing provinces in the west have lower rates of efficiency.

**Table 3:** Results of green governance efficiency.

	**Region**	**2008**	**2009**	**2010**	**2011**	**2012**	**2013**	**2014**	**2015**	**2016**	**2017**	**2018**	**Mean**
Eastern	Beijing	0.87	0.92	0.95	1.05	1.07	1.19	1.25	1.33	1.43	1.50	1.54	1.19
	Fujian	0.60	0.64	0.68	0.70	0.72	0.75	0.78	0.83	0.89	0.93	0.98	0.77
	Guangdong	0.70	0.73	0.74	0.75	0.80	0.83	0.87	0.90	0.93	0.98	0.99	0.84
	Hainan	0.61	0.66	0.69	0.71	0.75	0.77	0.82	0.85	0.89	0.91	0.93	0.78
	Hebei	0.46	0.47	0.50	0.51	0.52	0.62	0.70	0.75	0.76	0.78	0.82	0.63
	Jiangsu	0.71	0.73	0.71	0.76	0.79	0.75	0.82	0.84	0.88	0.90	0.95	0.80
	Liaoning	0.42	0.45	0.46	0.50	0.51	0.57	0.57	0.60	0.62	0.65	0.67	0.55
	Shandong	0.46	0.49	0.51	0.52	0.56	0.58	0.61	0.63	0.65	0.70	0.71	0.58
	Shanghai	0.75	0.77	0.78	0.83	0.87	0.92	0.94	1.11	1.12	1.14	1.18	0.95
	Tianjin	0.52	0.56	0.58	0.61	0.63	0.67	0.71	0.83	0.84	0.85	0.86	0.70
	Zhejiang	0.57	0.61	0.65	0.68	0.70	0.74	0.76	0.78	0.78	0.80	0.85	0.72
Eastern mean		0.61	0.64	0.66	0.69	0.72	0.76	0.80	0.86	0.89	0.92	0.95	0.77
Central	Anhui	0.37	0.38	0.40	0.41	0.42	0.43	0.45	0.46	0.49	0.51	0.53	0.44
	Heilongjiang	0.38	0.40	0.42	0.44	0.46	0.47	0.49	0.50	0.51	0.54	0.55	0.47
	Henan	0.39	0.41	0.42	0.44	0.44	0.46	0.48	0.50	0.51	0.52	0.55	0.47
	Hubei	0.65	0.66	0.68	0.70	0.72	0.77	0.80	0.82	0.85	0.87	0.89	0.76
	Hunan	0.61	0.63	0.65	0.67	0.70	0.76	0.79	0.84	0.85	0.87	0.97	0.76
	Jiangxi	0.34	0.35	0.36	0.38	0.39	0.40	0.43	0.45	0.46	0.47	0.50	0.41
	Jilin	0.33	0.34	0.36	0.38	0.39	0.41	0.44	0.45	0.46	0.47	0.49	0.41
	Shanxi	0.23	0.24	0.25	0.27	0.29	0.31	0.32	0.32	0.38	0.38	0.39	0.31
Central mean		0.41	0.43	0.44	0.46	0.48	0.50	0.52	0.54	0.56	0.58	0.61	0.50
Western	Chongqing	0.34	0.36	0.36	0.37	0.39	0.40	0.41	0.43	0.45	0.46	0.48	0.41
	Gansu	0.35	0.36	0.36	0.37	0.38	0.38	0.39	0.40	0.40	0.41	0.42	0.38
	Guangxi	0.32	0.33	0.34	0.35	0.37	0.38	0.38	0.41	0.41	0.42	0.46	0.38
	Guizhou	0.22	0.23	0.24	0.26	0.28	0.30	0.31	0.32	0.34	0.36	0.37	0.29
	Neimenggu	0.23	0.24	0.26	0.27	0.28	0.30	0.35	0.37	0.38	0.39	0.41	0.32
	Ningxia	0.23	0.24	0.25	0.27	0.29	0.30	0.31	0.35	0.37	0.40	0.43	0.31
	Qinghai	0.21	0.22	0.22	0.23	0.24	0.28	0.31	0.33	0.34	0.34	0.36	0.28
	Shaanxi	0.30	0.31	0.33	0.34	0.35	0.36	0.37	0.38	0.40	0.41	0.42	0.36
	Sichuan	0.32	0.33	0.34	0.35	0.36	0.37	0.37	0.38	0.39	0.40	0.42	0.37
	Xinjiang	0.13	0.13	0.16	0.17	0.18	0.19	0.20	0.35	0.21	0.23	0.22	0.20
	Yunnan	0.28	0.29	0.31	0.32	0.33	0.33	0.35	0.37	0.37	0.39	0.40	0.34
Western mean		0.27	0.28	0.29	0.30	0.31	0.33	0.34	0.37	0.37	0.38	0.40	0.33
National mean		0.43	0.45	0.47	0.49	0.51	0.53	0.56	0.60	0.61	0.63	0.66	0.54

As can be seen in Table 3, the country as a whole had an green governance efficiency rating of between 0.20 and 1.21. Between 2008 and 2018, the overall level was relatively stable, with only a small increase, and the gap between different regions was wide, narrowing sequentially in the eastern, central, and western regions. East China had the highest efficiency, averaging 0.79 from 2008 to 2018. At 1.2 and 0.98, respectively, Beijing and Shanghai were always the most productive provinces in China during the study period. More than 0.60 was the average across the eastern region. Green governance efficiency rose steadily from 2008 to 2018, peaking at 0.98 in 2017 and dipping slightly in 2018, with the remaining years showing a slow upward trend. A value of 0.43–0.66 was the range for the central efficiency from 2008 to 2018. Over the period 2008–2018, there has been a slight rise in the overall trend. Though the central region's average efficiency was higher than the national average and lower than the eastern region's average efficiency, it increased the most since 2018, reaching a value of 0.66 in 2018. As a result, Shanxi had the lowest average efficiency value of any province in China, at just 0.31.

There was a narrowest range of change in the western region's value from 2007 to 2019. The efficiency value ranged from 0.268 to 0.349 and was steadily rising. Despite this, West China's green governance efficiency was the worst in the country, and even worse than that of the eastern region. green governance efficiency inefficient provinces in this region include Xinjiang, Ningxia, Guizhou and Qinghai. In general, China's energy efficiency is highly variable. From west to east, the spatial gradient increases, with many regions with the lowest efficiency residing in West China, and regions with the highest or second-highest efficiency mainly located in East China. This is consistent with the distribution of economic power in China, according to this study.

### Econometric Estimation

#### Cross-Sectional Dependence Test Results

The long-term model estimate is the first step in the empirical evaluation. To choose an appropriate method for long-term assessment, the cross-sectional dependency must be examined. The subject of today's literature is the evaluation of CD tests. Inability to handle the CD may result in only partial results. The CD's outcomes are revealed in Table 3. At 1%, the outcomes are significant and show that the null hypothesis is rejected. The CD's existence is confirmed by the results in Table 4.

**Table 4:** Test results of cross-sectional dependence.

**Variable**	**CD test**	***p*-value**	**Corr**
CO_2_	6.446	0.000	0.123
GGEI	36.922	0.000	0.706
FI	39.065	0.000	0.747
GF	29.186	0.000	0.558
NR	45.712	0.000	0.874
HC	0.184	0.858	0.003
RMT	25.344	0.000	0.023

Through unit root assessments, the heterogeneity or occurrence of the cross sectional dependence test permits for the examination of the integration order of the variables in the second generation. As a result, the root unit tests CIPS and CADF are applied, and the outcomes of both tests are summarized in Table 5. The variables are not stationary, according to the null hypothesis CIPS and CADF.

**Table 5:** Unit root methods result.

	**CIPS**		**CADF**	
	**Level**	**First difference**	**Level**	**First difference**
CO_2_	−1.358	−2.641***	−1.828	−2.627***
GGEI	−1.489	−2.656***	−1.581	−2.416***
FI	−2.263	−2.431***	−2.052	−2.321***
GF	−2.406	−3.180***	−2.230	−3.252***
NR	−1.589	−2.147***	−1.841	−2.740***
HC	−1.837	−2.335***	−2.406	−2.189***
RMT	−1.643	−2.515***	−2.596	−2.526***

Significance is indicated by 10, 5, and 1% though *, **, and ***.

#### Model Comparison

To compare the results of three different models with the panel quantile regression model, the study estimates three conditional mean (CM) regression models. The results of three different model are given in Table 6. The autoregressive AR2 estimate outcomes disclose that the random error term has no second-order sequence correlation. The coefficients of green governance, green finance, and financial inclusion are negative and significant at the 1% level, according to the regression results. It demonstrates green governance, green finance and financial inclusion have a “U-shaped” relationship. To put it another way, even before the degree of green governance, green finance, and financial inclusion reaches a tipping point, an increase in green governance, green finance, and financial inclusion will reduce environmental pollution (67). However, a moderate improvement in green governance, green finance and financial inclusion can help to improve sustainable development. The following things are to blame for this. There is a lack of incentive for companies to implement green technology innovations to reduce emissions and conserve energy when environmental regulation is lax because environmental payment costs make up a small part of total expenditures for companies.

**Table 6:** Model comparison.

**Variables**	**OLS pooled**	**OLS One-way fixed effect**	**OLS two-way fixed effect**
GGE	−0.763***	−0.743***	−0.785***
	(0.374)	(0.374)	(0.374)
GF	−0.051***	−0.121***	−0.122***
	(−2.532)	(−3.452)	(−4.106)
FI	−0.011**	−0.048***	−0.045***
	(−2.213)	(−3.841)	(−3.591)
NR	0.312***	0.223	0.102
	(3.132)	(1.466)	(0.684)
HC	0.050**	0.061	0.069
	(2.301)	(0.059)	(1.175)
RMT	−0.035***	−0.010***	−0.009***
	(−11.221)	(−4.093)	(−3.653)
Constant	5.252***	5.830***	5.434***
	(2.051)	(1.362)	(1.597)
R2		0.904	0.908
F/Wald test	153.15***	22.00***	178.27***

Significance is indicated by 10, 5, and 1% though *, **, and ***.

#### Panel Quantile Regression

The panel quantile regression estimators of Koenker and Bassett (68) are used to forecast the long-term coefficients. Table 7 summarizes the regression analysis' relevant findings. CO_2_ emissions in rising economies are expected to rank 10th, 20th, 30th, 40th, 50th, 60th, 70th, 80th, and 90th, respectively.

**Table 7:** Test results of panel quantile regression.

**Variables**	**Q0.1**	**Q0.2**	**Q0.3**	**Q0.4**	**Q0.5**	**Q0.6**	**Q0.7**	**Q0.8**	**Q0.9**
GGE	0.343^**^	0.419^*^	0.329^*^	0.276^*^	0.326^*^	0.331^*^	0.414^*^	0.581^*^	0.527^*^
FI	0.199^*^	0.049^*^	−0. 085^**^	−0 0.043^**^	−0. 066^**^	−0. 090^*^	−0. 127^**^	−0. 168^*^	−0.153^*^
GF	−0. 057^*^	−0. 188	−0. 468^**^	−0.556^*^	−0. 566^*^	−0. 599^*^	−0. 459^*^	−0. 312^**^	−0.539^*^
NR	0.0167^***^	0.062	0.085	0.094^*^	0.097^*^	0.102^*^	00,892^**^	0.029^**^	0.026^*^
HC	0.021	0.034	0.038^*^	0.048^*^	0.047^*^	0.052^*^	0.015^*^	−0. 043^*^	0.036^*^
RMT	−0. 021^**^	−0. 034^*^	−0. 038^*^	−0. 048^*^	−0. 047^*^	−0. 052^*^	−0.015^*^	−0. 043^*^	−0.036^*^

* *Significant value at 1%*,

** *significant value at 5%, and*

*** *denote significant value at 10%*.

##### Green Governance Efficiency

In all quantiles, the PQR findings reveal a negative and statistically significant link between green governance and CO_2_ emissions except lower quantiles. The results of the study show that the carbon dioxide emission levels are higher in provinces with lower green governance efficiency. Furthermore, from the middle to the upper quantiles, the impact of green governance on CO_2_ emissions increases. It indicates that efficient green governance reduces emissions in these provinces. Our findings are consist with the prior studies (69–72).

##### Green Finance

The findings shows that in all quantiles green finance has positive and statistically significant relationship with CO_2_ emissions. Environmental pollution will be reduced in the long run if green finance policy is implemented, according to the experimental results. So, how can environmental pollution be reduced as a result of green financing? Environmental improvement can be achieved if green finance policies are put into place (73), which will restrict businesses' frequent pollution behaviors and encourage more capital to flow into low-pollution industries.

##### Financial Inclusion

Except for the Q0.1 and Q0.2 quantile, the outcomes indicates that financial inclusion has a negative and statistically significant influence on CO_2_ emissions. This results proposes that as financial inclusion rises, pollution rises with it. The positive effect could be due to rising financial inclusion, which permits customers to buying energy intensive household machines such as refrigerators, air conditioners, and automobiles, as more CO_2_ emissions are emitted, this has significant ecological impacts. Moreover, after the first quantile, findings indicate that there negative and statistically significant correlation between financial inclusion and CO_2_ emissions in all quantiles. It could be argued that, following the abolition of lending amounts in society and good money management could result in a reduction in carbon emissions. Modern technology, which is the only way to reduce carbon emissions, is always required for efficient money management. Our findings and argument differ from those of (51), they argue that increasing financial inclusion is a factor in high pollution rates. Our findings, on the other hand, suggest that greater financial inclusion could lead to increased use of renewable energy, which is good for the sustainable development. Financial inclusion can help decrease the negative environmental impact of economic growth and restore environmental welfare.

The study also includes Control variables, to better understand the impact on environmental quality.

##### Natural Resources

The coefficient of natural resources estimates, on the other hand, show that natural resources can be a reliable way to improve the environmental quality of China. The effect of natural resources on CO_2_ emissions is positive and statistically significant for the 40th, to 90th quantiles, but at the 20th and 30th quantiles, the effect is statistically insignificant and positive. As a result, the use of natural resources has a negative impact on the environment, implying that countries that rely heavily on unsustainable natural resources should increase their imports of filthy energy sources (51).

##### Human Capital

Table 7 also reveals that human capital has no impact on CO_2_ emissions in the first two quantiles, but has a positive and significant impact in the remaining quantiles. However, while human capital is now growing its emissions, it also has inherent environmental skills that may be exploited to protect the environment. While Adedoyin et al. (74) discovered that the level of human capital affects economic development's effect on emissions, this study finds that as human capital increases, so does environmental awareness and environmentally friendly technologies.

##### Remittances

The PQR findings also show a negative relationship between remittances and carbon emissions. When foreign inflows increase, modern technology enters the country, which can help to reduce emissions. It demonstrates that remittances reduce emissions indirectly by affecting financial inclusion. In the low, medium, and high quantiles, remittances have the same negative impact on CO_2_ emissions. On all quantiles, the results show a positive influence of economic growth on carbon emissions. It is an indication that increased economic activity accelerates CO_2_ emissions, which are primarily due to the use of non-renewable energy. Because economic growth improves the country's level of financial inclusion, we used GDP as a control variable in our research.

### Long-Run Panel Cointegration

Our main findings using the quantile regression method show that green governance efficiency, green finance, and financial inclusion have a negative and significant influence on CO_2_ emissions, while GDPpc, human capital, and remittances have a positive impact. We use two more regression tests, Cup-BC and Cup-FM, to check the robustness of our empirical results (see Table 8). Green governance has negative relationship with carbon emissions, according to the findings. Green governance reduces CO_2_ emissions by 0.832% in China. Financial inclusion has a 1% significant negative impact on carbon emissions, according to the data. This means that a 1% increase in financial inclusion in China results in a 0.078% reduce in CO_2_ emissions. In terms of the impact of green finance, the findings show a negative 1% significant ratio of CO_2_ emissions from green finance. As a result, a 1% increase in green finance in reduces CO_2_ emissions by 0.089%. Cup-FM and Cup-BC coefficients are similar to our initial quantile regression results, according to our findings. Since then, we've come to the conclusion that our preliminary findings on the impact of financial inclusion, human capital, and natural resources on CO_2_ emissions are reliable.

**Table 8:** Panel regression test results.

**Variable**	**Cup-FM**		**Cup-BC**	
	**Coefficient**	***t*-statistics**	**Coefficient**	***t*-statistics**
GGE	−0.832*	13.566	−0.832*	13.567
FI	0.078*	3.770	0.084*	4.138
GF	−0.089*	2.289	−0.078*	2.177
NR	0.131*	7.081	0.153*	6.429
HC	0.041*	3.092	0.040*	3.186
RMT	−0.288*	2.636	−0.032*	3.193

Significance is indicated by 10, 5, and 1% though *, **, and ***.

## Conclusions and Policy Implication

By its nature, green governance is dynamic. Many countries formerly classified as developing or poor have made significant progress in environmental enforcement and environmental protection legislation over the course of decades of continuous efforts. This study Based on the connotation of green governance, this paper looks at how China is previously applying a number of green governance and green finance policy initiatives, as well as how these initiatives have influenced sustainable development over time. This paper constructs a generalized panel Three-stage DEA model to calculate the green governance index of 30 Chinese provinces from 2008 to 2018. The findings clarifies the direction and optimization of green governance efficiency through the external environment. This paper uses the panel data model to investigate the impact of green governance, green finance, and financial inclusion on sustainable development. The econometric model findings demonstrated that overall green governance and green finance policies result in positive environmental outcomes. The research then examines the character of financial inclusion in environmental protection. According to our groundbreaking results, green governance efficiency contributes to lower CO_2_ emissions and complements environmental security investment initiatives.

### Policy Implication

The following policy implications emerge as a result of this discovery:

1. The government should properly control energy markets and prevent extreme intervention in energy pricing in underdeveloped regions with substantial energy price distortions. Meanwhile, attempts to minimize the outflow of local energy advantages, promote the free movement of energy components across areas, and diminish the geographical agglomeration of energy pricing distortions should weaken market segmentation.
2. Energy price strategies for different regions should be implemented. Energy pricing should be moved from the government to the market in industrialized regions with low energy price distortions, so that energy input can reveal profits that balance its worth.
3. The energy price changes should be pushed at the same time as advanced technology advancements and energy consumption mix optimization.
4. To successfully improve environmental quality, businesses should raise investment in scientific research, accelerate technical innovation, and enhance the development and exploitation of clean industrial technologies. Simultaneously, they should raise the proportion of clean energy in overall energy consumption by speeding up the optimization and upgrading of the energy consumption structure.
5. Green finance products must be developed more quickly, and financial institutions' ability to provide green credit must be strengthened.
6. It is necessary to invest more in basic study on how to implement green finance products while minimizing associated risks.
7. There should be incentives for green finance and environmental protection activities to promote green consumption and regulators should limit the systemic risk of fintechs.

### Research Limitations

There are a couple of caveats to our findings. We were unable to investigate all of the various factors that influence our research questions, considering long-term consequences, due to a lack of data. Due to a lack of appropriate instrumental variables, we were unable to address endogeneity and simultaneity issues. Despite the limitations mentioned above, this paper provides useful information on how green finance and fintech development can contribute to environmental protection and long-term growth.

## Data Availability Statement

The raw data supporting the conclusions of this article will be made available by the authors, without undue reservation.

## Author Contributions

All authors listed have made a substantial, direct, and intellectual contribution to the work and approved it for publication.

## Funding

This study was supported by the Hebei Financial Innovation and Risk Management Research Center Open Fund Project (No. 2017JDKF021).

## Conflict of Interest

The authors declare that the research was conducted in the absence of any commercial or financial relationships that could be construed as a potential conflict of interest.

## Publisher's Note

All claims expressed in this article are solely those of the authors and do not necessarily represent those of their affiliated organizations, or those of the publisher, the editors and the reviewers. Any product that may be evaluated in this article, or claim that may be made by its manufacturer, is not guaranteed or endorsed by the publisher.
